# Cytokines and olfactory bulb microglia in response to bacterial challenge in the compromised primary olfactory pathway

**DOI:** 10.1186/1742-2094-9-109

**Published:** 2012-05-29

**Authors:** Rosalind P Herbert, Julie Harris, Kim Pei Chong, Jamie Chapman, Adrian K West, Meng Inn Chuah

**Affiliations:** 1 Menzies Research Institute Tasmania, 17 Liverpool Street, Hobart, TAS, 7001, Australia; 2 School of Medicine, The University of Tasmania, 17 Liverpool Street, Hobart, TAS, 7001, Australia

**Keywords:** Olfactory mucosa, Innate immunity, Cytokines, Bacterial infection, Microglia, Olfactory ensheathing cells

## Abstract

**Background:**

The primary olfactory pathway is a potential route through which microorganisms from the periphery could potentially access the central nervous system. Our previous studies demonstrated that if the olfactory epithelium was damaged, bacteria administered into the nasal cavity induced nitric oxide production in olfactory ensheathing cells. This study investigates the cytokine profile of olfactory tissues as a consequence of bacterial challenge and establishes whether or not the bacteria are able to reach the olfactory bulb in the central nervous system.

**Methods:**

The olfactory epithelium of C57BL/6 mice was damaged by unilateral Triton X-100 nasal washing, and *Staphylococcus aureus* was administered ipsilaterally 4 days later. Olfactory mucosa and bulb were harvested 6 h, 24 h and 5 days after inoculation and their cytokine profile compared to control tissues. The fate of *S. aureus* and the response of bulbar microglia were examined using fluorescence microscopy and transmission electron microscopy.

**Results:**

In the olfactory mucosa, administered *S. aureus* was present in supporting cells of the olfactory epithelium, and macrophages and olfactory nerve bundles in the lamina propria. Fluorescein isothiocyanate-conjugated *S. aureus* was observed within the olfactory mucosa and bulb 6 h after inoculation, but remained restricted to the peripheral layers up to 5 days later. At the 24-h time point, the level of interleukin-6 (IL-6) and tumour necrosis factor-α in the compromised olfactory tissues challenged with bacteria (12,466 ± 956 pg/ml and 552 ± 193 pg/ml, respectively) was significantly higher than that in compromised olfactory tissues alone (6,092 ± 1,403 pg/ml and 80 ± 2 pg/ml, respectively). Immunohistochemistry confirmed that IL-6 was present in several cell types including olfactory ensheathing cells and mitral cells of the olfactory bulb. Concurrently, there was a 4.4-, 4.5- and 2.8-fold increase in the density of iNOS-expressing cells in the olfactory mucosa, olfactory nerve and glomerular layers combined, and granule layer of the olfactory bulb, respectively.

**Conclusions:**

Bacteria are able to penetrate the immunological defence of the compromised olfactory mucosa and infiltrate the olfactory bulb within 6 h even though a proinflammatory profile is mounted. Activated microglia may have a role in restricting bacteria to the outer layers of the olfactory bulb.

## Background

The primary olfactory pathway represents a one-synapse route spanning the olfactory mucosa and the olfactory bulb. Normally, microbes are prevented from infecting the olfactory mucosa through sneezing, clearing of mucous, immune responses by cells in the nasal mucosa or apoptosis of infected cells [1,2]. Though generally successful, these protective strategies are known to fail under certain conditions. Viruses injected into the nasal cavity have been found to enter olfactory neurons, replicate, travel into the olfactory bulb and then into the central nervous system (CNS) [3,4]. In addition, viruses in circulation can also cross from capillaries in the lamina propria into olfactory neurons. Because of the close proximity and the synaptic connections between the nasal cavity and the CNS, the brain can potentially be infected in relatively short periods of time [3]. Ginkel and co-workers demonstrated that intranasal injection of a strain of *Streptococcus pneumonia*, which is non-viable in the blood system, resulted in olfactory bulb and CNS infection *via* olfactory axonal transport after just 24 h [5]. The infection of olfactory neurons was thought to be through pneumococci interaction with gangliosides expressed on the cell membranes. The rarity of these infections suggests that a robust endogenous immunological defence is likely to be present in olfactory tissues, but this immunological response has been poorly studied.

In a recent study, we showed that the intact olfactory epithelium was an effective barrier in preventing the infiltration of *Staphylococcus aureus* into the nasal lamina propria [6]. However if the olfactory epithelium was damaged by zinc sulphate or Triton-X irrigation, it was susceptible to bacterial infiltration, and this led to the appearance of inducible nitric oxide synthase (iNOS)-expressing cells in the lamina propria, some of which were the neuroglia of olfactory nerves, the olfactory ensheathing cells (OECs) [6]. Consistent with this finding, nitric oxide production by OECS was demonstrated within 1 h of bacterial exposure *in vitro*[6]. Furthermore, microarray analysis showed that OECs have enriched transcripts for the chemokines CXCL1 and MCP-1 [7], both of which increased when OECs were exposed to *Escherichia coli* and *S. aureus in vitro*[8]. CX_3_CL1, a cytokine known to influence the migration of CX_3_CR1-positive innate immune cells into tissue [9], is also expressed by olfactory neurons and OECs [10]. Thus, the evidence suggests the existence of an immune barrier in the nasal cavity that is contributed in part by OECs.

This study aims to build on these previous findings and obtain new knowledge about the immunological status of the olfactory mucosa and bulb. We investigated the cytokine and macrophage profile of damaged olfactory tissues in response to bacterial challenge and tracked the infiltration of bacteria into the olfactory bulb.

## Methods

All experiments involving animals in this project were approved by the Animal Experimentation Ethics Committee of the University of Tasmania and are consistent with the Australian Code of Practice for the Care and Use of Animals for Scientific Purpose. mice aged 6–7 weeks were maintained on a 12-h light:dark cycle in filtered, sterile air by the Menzies Research Institute Animal Services.

### S. Aureus cultures and fluorescein isothiocyanate (FITC) conjugation

A loopful from slope cultures of *S. aureus* (ATCC 33862) (kindly donated by the Microbiology Department of the University of Tasmania) was streaked onto nutrient agar plates and incubated overnight at 37^0^ C. Single colonies were then picked and inoculated into 1 ml L-broth cultures (pH 7.2) and incubated overnight at 37^0^ C, shaking at 300 rpm in an orbital shaker (Ratek Instruments, Australia). Bacteria from tubes that exhibited growth, as evidenced by turbidity of broth, were inoculated directly into the left nasal cavity of mice in the appropriate treatment group at the designated time points.

**Figure 1:**
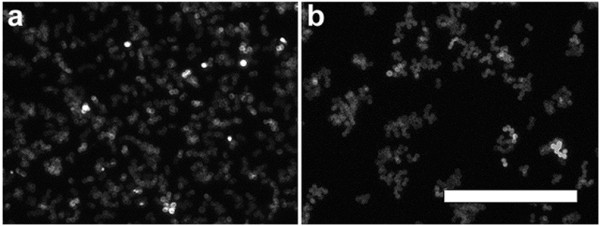
***S. aureus*conjugated with FITC prior to inoculation in the nasal cavity (A);*S. aureus*conjugated with FITC and cultured on nutrient agar plates overnight, confirming viability and continued fluorescence (B).*Scale bar*** **= 10 μm.**

In order to visually localise bacteria under fluorescence microscopy, *S. aureus* was conjugated to FITC by incubating 10 μl FITC (1 mg/ml; Sigma Aldrich, Australia) in 1 ml L-broth and *S. aureus* at 37^0^ C for 1.5 h. The culture was then washed with 500 μl filtered PBS and centrifuged at 12,000 rpm for 30 s. The supernatant was removed, and the pellet washed twice and resuspended in 200 μl filtered PBS prior to nasal inoculation. Following FITC conjugation, random samples of *S. aureus* were checked on the fluorescence microscope to confirm that conjugation was successful (Figure 1A). In addition, some were plated on nutrient agar plates and left overnight at 37^0^ C. Growth was typically observed, indicating viable bacteria as well as continued fluorescence (Figure 1B).

### Cytokine assay of olfactory tissues

Animals were divided into four groups: (1) untreated mice (NT); (2) mice treated with 30 μl 1% Triton X-100 alone (TX); (3) mice treated with 30 μl 1% Triton X-100 and 30 μl 0.9% sterile saline 4 days later (TXS); (4) mice treated with 30 μl Triton X-100 and 30 μl *S. aureus* 4 days later (TXB). The amount of *S. aureus* inoculated into the nasal cavity was equivalent to 3 x 10^7^*.* Bacteria were introduced 4 days after Triton X-100 washing because at this time most of the damaged olfactory neurons have sloughed off while the basal cells have initiated proliferation to reconstitute the epithelium [11]. Nasal irrigation was performed unilaterally on the left side of the nasal cavity using 30-gauge plastic tubing attached to the end of a 29-G needle. Mice were lightly anaesthetised with 0.6% isoflurane in air prior to Triton X-100, saline or *S. aureus* inoculation.

Mice of the TX group were perfused 4 days after Triton X-100 irrigation; mice of the TXS and TXB groups were perfused either 6 h (TXS6, TXB6 respectively) or 24 h (TXS24, TXB24 respectively) after saline or *S. aureus* inoculation. Mice were euthanised with sodium pentobarbitone (50 mg/kg i.p.) before transcardial perfusion with chilled phosphate-buffered saline (PBS) in an aseptic environment. The olfactory bulbs, olfactory turbinates and septum were dissected. Tissue from each group was pooled, minced and homogenised in lysis buffer (catalogue no. 0103004-L; RayBioTech) with 3% protease inhibitor. Samples were centrifuged at 13,000 g for 10 min at 4 °C. The supernatant and pellet were separated, snap frozen in liquid nitrogen and stored at −80 °C until use in cytokine assay.

A modified Bradford assay [12] was carried out to establish the total protein concentration of the pooled olfactory tissue supernatant from each treatment group. This was done in order to estimate the correct sample amount to be used in the cytokine arrays. One part Bradford reagent was diluted for four parts distilled water. Standard protein samples at concentrations of 0.1, 0.15, 0.2, 0.25, 0.5 and 1.0 mg/ml were prepared using DNase free bovine serum albumin. Each standard was diluted 1:50 using Bradford reagent in a 96-well plate. Samples were diluted 1:100 in MilliQ water and then further diluted 1:100 in a 96-well plate using Bradford reagent. Replicates were made of each standard and sample. Absorbance at 595 nm was measured using a Tecan GENios microplate reader and analysed using the XFluor4 (v.4.5) software. From measured absorbance of standards, a standard curve was created and used to determine the concentration of the samples.

Cytokine assay was performed as three separate experiments for each time point. For each time point, tissue was pooled from three untreated mice and four mice from each of the treated groups (TX, TXS and TXB) and samples diluted to a concentration of 25 mg/ml. Cytometric Bead Array Mouse Th1/Th2/Th17 Cytokine Kit (BD Biosciences) detects seven cytokines (interferon-γ, interleukin-10, interleukin-17A, interleukin-2, interleukin-4, interleukin-6, tumour necrosis factor-α) using flow cytometry. The assay was run according to the manufacturer’s instructions. Replicates were run on a BD FACS Canto II flow cytometer with 2,100 events measured in the P1 gate, and total events were also recorded. FCAP array (v1.0.1) software was used to analyse data collected from the flow cytometer. Raw data from the BD array were multiplied by the appropriate dilution factor to calculate the true concentration of cytokines. Cytokine concentrations for the BD array were tabulated, and data analyses were conducted using single factor ANOVA and pairwise multiple comparison (Tukey test). A *p* value equal to or less than 0.05 was considered statistically significant.

### Visualisation of FITC-conjugated S. aureus and immunohistochemical staining for interleukin-6 (IL-6)

A total of 30 μl of *S. aureus* was inoculated into the left nasal cavity of eight 6–7-week-old C57/BL6 male mice that had previously undergone unilateral nasal washing with 1% Triton-X 100 solution. Mice were killed at 6 h (*n* = 3), 24 h (*n* = 2) and 5 days (*n* = 3) after inoculation. Transcardial perfusion was conducted with chilled PBS followed by Zamboni’s fixative (ZF; 15% picric acid + 0.85% paraformaldehyde). Mice were decapitated and excess tissue trimmed away, leaving the olfactory bulbs and nasal cavity en bloc; they were then stored in ZF at 4 °C. Tissue was washed with PBS several times after 2–3 days to remove ZF before being transferred to 0.01% azide in PBS. The nasal cavity and olfactory bulb en bloc were decalcified in clinical fast decal fluid (Lomb Scientific) for 1–1.5 h. Specimens were washed with distilled water, trimmed and cryoprotected by transferring to 10% sucrose overnight, followed by 30% sucrose. They were then frozen in cryomatrix, sectioned at 10 μm in the horizontal plane using a cryostat (Leica DM-1850), placed onto SuperFrost Plus slides (Menzel Glaser, Germany) and stored at -20^0^ C. Unstained sections from all animals were examined on an Olympus BX50 fluorescent microscope to localise FITC-conjugated *S. aureus*.

For immunohistochemical detection of IL-6, sections were post-fixed with 4% paraformaldehyde for 5 min, washed for 5 min in PBS and then incubated with 10% hydrogen peroxide to quench endogenous peroxidase. Slides were then incubated with Mouse on Mouse blocking reagent from the Vector M.O.M Kit for 1 h. They were then incubated with Mouse on Mouse diluent from the M.O.M Kit for 5 min. Sections were incubated with primary antibody rat anti- IL-6 (1:100; Invitrogen) overnight at 4^0^ C. Control sections were incubated overnight with diluent alone or isotype control. Next, sections were incubated with biotinylated goat anti-rat antibody (1:1;000, Molecular Probes) for 1 h followed by incubation with streptavidin conjugated to HRP for 30 min, and DAB-chromogen solution for 5 to 6 min. Sections were counterstained with Nuclear Red in 0.03% azide for 1.5 min, coverslipped and viewed on a Leica DM-2500 light microscope. Control sections did not show positive immunoreactivity.

### Olfactory ensheathing cell culture and immunostaining for IL-6

Olfactory ensheathing cells were isolated from the olfactory nerve layer of the olfactory bulb and the olfactory mucosa of 3-day-old mouse pups as described in a previous study [13]. Briefly, the olfactory bulbs and mucosa were dissected and digested for 20 min using 0.25% trypsin in MEM-H (Gibco) at 37 °C. Dulbecco’s modified Eagle’s medium with HEPES (DMEM-H; Gibco) supplemented with 10% foetal calf serum (DMEM-10FCS) was added to terminate digestion, and the suspension was centrifuged at 500 g for 10 min. The supernatant was removed and the pellet resuspended in DMEM-H. Cells were dissociated and plated onto acid-washed 13-mm coverslips coated with 4% poly-L-lysine. Following incubation for 24 h at 37 °C, cytosine-β-D-arabinofuranoside (100 μM) was added and cells were incubated for another 2 days at 37 °C until cultures were sufficiently free of contaminating cells. The medium was then replaced with fresh DMEM- 10FCS supplemented with bovine pituitary extract (50 μg/ml; Sigma) to allow cellular expansion. Cultures yielded 95% OECs based on positive immunoreactivity with anti-p75NTR.

The immunostaining procedure for IL-6 was similar to that used on tissues sections, except that cultures were treated with a shorter incubation time (1 h) with the primary antibody. Cells were counterstained with Nuclear Red in 0.03% azide for 1 min, dehydrated and mounted with DPX.

### Immunofluorescent staining with Iba1, anti-iNOS and tomato lectin, and quantitative analysis

The left nasal cavity of six 6–7-week-old C57/BL6 male mice that had previously undergone unilateral nasal washing with 1% Triton-X 100 solution was inoculated with 30 μl of *S. aureus* (3 mice) or saline (3 mice). The mice were euthanised with sodium pentobarbitone (50 mg/kg i.p.) at 24 h after bacterial inoculation and transcardially perfused with chilled PBS followed by 4% paraformaldehyde. For comparison, three untreated 6–7-week-old C57/BL6 male mice were also killed and transcardially perfused. Mice were decapitated, and the heads trimmed and processed for cryosectioning, as described for immunohistochemistry in the preceding section. Ten-micron-thick sections were post-fixed with 4% paraformaldehyde for 15 min, washed gently three times with PBS and blocked using Dako Protein Block Serum-free (DakoCytomation) for 5 min prior to incubation with primary antibodies. Microglia were identified by positive immunoreactivity for Iba1 or binding to tomato lectin [14,15]. In single labelling, sections were incubated with rabbit-anti iNOS/NOS type II (1:400; BD Biosciences), Iba1 (1:200; Wako) or biotinylated tomato lectin (10 μg/ml diluted 1:200; Vector Labs) for 2 h. This was followed by incubation with goat anti-rabbit AlexaFluor 594 (1:1,000; Molecular Probes), goat anti-mouse AlexaFluor 488 (1:1000; Molecular Probes) or streptavidin AlexaFluor 488 (1:1000; Molecular Probes) respectively for 1 h at room temperature in the dark, followed by 0.0001% Nuclear Yellow (Sigma) staining for 5 min prior to mounting and storage at 4^0^ C. In some sections, double labelling with Iba1 and tomato lectin was done to ascertain colocalisation. Double labelling with anti- iNOS and tomato lectin was also done to identify iNOS-producing microglia.

Nine separate frames, taken with 40× objective, capturing the olfactory nerve and glomerular layers of the rostral part of the olfactory bulb from each animal were used in the cell counts of iNOS- and Iba1-positive cells. Cell counts and epithelial distance, as represented by the basal lamina, were measured using Image J. The data were analysed by two-way ANOVA and pairwise multiple comparison (Tukey test). A *p* value equal to or less than 0.05 was considered statistically significant.

### Transmission electron microscopy

Six mice were subjected to unilateral nasal wash with 1% Triton-X 100 solution and 4 days after received 30 μl of live *S. aureus* in the ipsilateral nasal cavity. Six and 12 h after bacterial inoculation, mice were perfused with chilled PBS followed by 2.5% glutaraldehyde in 0.2 M cacodylate buffer. The nasal septum was dissected, immersed in 2.5% glutaraldehyde overnight at 4^0^ C and processed according to methods established previously in our laboratory [16]. The following day specimens were washed in 0.1 M cacodylate buffer, post-fixed in 1% osmium tetroxide (2 h), rinsed in cacodylate buffer and stained in saturated uranyl acetate in 50% ethanol for 10 min. Next, specimens were washed and dehydrated in an increasing series of ethanols (70–100%). Specimens were cleared in three changes of propylene oxide and immersed overnight in a 1:4 mix of propylene oxide and Procure 812 resin. Specimens were then transferred to BEEM capsules and embedded in Procure 812 resin in a 60^0^ C oven for 48 h. Thick (1 μm) and ultrathin (70–90 nm) sections were cut using a Leica ultramicrotome and placed on formvar-coated copper palladium grids (ProSciTech, Qld). Thin sections were stained with saturated uranyl acetate in 5% ethanol (30 min) followed by lead citrate (5 min) prior to being viewed on a Philips CM100 transmission electron microscope.

## Results

### Olfactory tissues damaged by Triton-X 100 significantly upregulate the expression of IL-6 and TNF-α when exposed to bacteria

**Figure 2:**
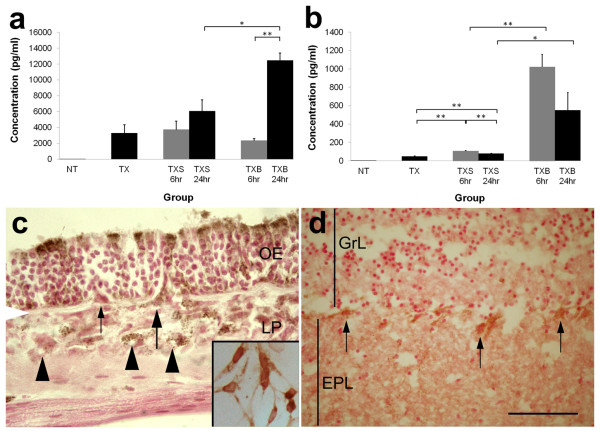
**Measurement of interleukin-6 (IL-6; A) and tumour necrosis factor-α (TNF-α; B) in groups of mice: untreated (*NT*); unilateral nasal wash with Triton X-100 alone (*TX*); unilateral nasal wash with Triton X-100 followed by saline wash (*TXS*); unilateral nasal wash with Triton X-100 followed by*S. aureus*inoculation (*TXB*).** Undamaged normal olfactory tissues express negligible amounts of IL-6 and TNF-α. At 4 days post-Triton X-100 damage (*TX*), olfactory tissues had an initial expression of IL-6 and TNF-α of 3,309 ± 1,052 pg/ml and 49 ± 6 pg/ml respectively. The level of IL-6 is significantly increased by the presence of bacteria at the 24-h time point (12,466 ± 956 pg/ml). The upregulation of IL-6 appears to follow that of TNF-α, which occurs at the 6-h time point, albeit at a lower concentration (1,024 ± 137 pg/ml). Thereafter, at the 24-h time point, a decreasing trend in TNF-α production is observed. *Error bars* represent SEM. *p < 0.05; **p < 0.01. IL-6 expression in the olfactory mucosa (**C**) and olfactory bulb (**D**) 24 h post-inoculation with *S. aureus.* IL-6 immunoreactivity is present in the cytoplasm of select groups of supporting cells in the apical part of the olfactory epithelium (*OE*). Some cells straddling the OE and lamina propria (*LP*), possibly OECs or macrophages, express IL-6 (*large arrow*), while others do not (*small arrow*). Several cells in the LP are immunopositive for IL-6 including some that surround the small lumen (*arrowheads*), which are most likely endothelial cells or inflammatory cells. *Inset* shows immunoreactivity for IL-6 in cultured OECs. *White arrowhead* at the edge of the micrograph indicates location of basal lamina. In the olfactory bulb mitral cell soma (*arrows*) show positive immunostaining for IL-6 with more diffuse staining in the external plexiform layer (*EPL*). *GrL* = granule cell layer; *scale bar* = 40 μm (*inset* in **C**); 50 μm (**C,D**).

Triton X-100 was used in this study because its cellular effects are well characterised [11,17]. It selectively destroys mature olfactory neurons and leaves the rest of the olfactory mucosa intact with no direct damage to the olfactory bulb. In the normal olfactory mucosa and bulb there was negligible expression of IL-6 (26 ± 7 pg/ml) (Figure 2A). Olfactory epithelial ablation by Triton-X 100 induced expression of IL-6, which was measured 4 days later (3,309 ± 1,052 pg/ml; *p* < 0.01). When this damaged olfactory mucosa was exposed to *S. aureus*, a further significant upregulation of IL-6 was observed 24 h later (12,466 ± 956 pg/ml). This was also significantly higher than that of similarly ablated olfactory tissues subjected to saline wash at the 24-h time point (6,092 ± 1,403 pg/ml; *p* < 0.05). No significant difference between these two treatment groups (*p* = 0.26) was detected at the 6-h time point. There was no significant difference in IL-6 concentration between the TX (3,309 ± 1,052 pg/ml) and TXS groups (6 h = 3,748 ± 1,086 pg/ml, *p* = 0.80; 24 h = 6,092 ± 1,403 pg/ml, *p* = 0.15), suggesting that nasal washing *per se* did not induce IL-6 upregulation.

Immunohistochemical staining showed that IL-6 was present in the apical region of sustentacular cells in the olfactory epithelium, as well as in cells throughout the lamina propria (Figure 2C), some of which could be OECs or infiltrating immune cells. IL-6-positive cells that straddled the olfactory epithelium and the lamina propria are consistent with OECs as they emerge from the olfactory epithelium to accompany differentiating axons [18]. Alternatively they could be macrophages that reside in the olfactory epithelium [10]. Other IL-6-positive cells within the lamina propria appeared to surround blood vessels, and these cells could either be endothelial cells or infiltrating inflammatory cells. Subsequent isolation of OECs from the olfactory mucosa and bulb, and cell culture confirmed that OECs expressed IL-6 (inset in Figure 2C). In the olfactory bulb, immunostaining for IL-6 was observed in the external plexiform layer and mitral cells (Figure 2D) and less intensely in the glomerular and olfactory nerve layer.

At 4 days post-injury with Triton X-100, olfactory tissues showed an initial level of 49 ± 6 pg/ml TNF-α, which increased significantly to 1,024 1 ± 37 pg/ml 6 h after *S. aureus* exposure (*p* < 0.01) (Figure 2B). In comparison, Triton X-100 damaged olfactory tissues exposed to saline alone yielded 108 ± 4 pg/ml TNF-α. There was no significant difference (*p* = 0.10) in the TNF-α response to bacteria between 6 and 24 h (552 ± 193 pg/ml), although there appeared to be a downward trend. TNF-α concentration at both 6 and 24 h after *S. aureus* inoculation remained significantly higher when compared to the saline control (6 h = 108 ± 4 pg/ml, *p* < 0.01; 24 h = 80 ± 2 pg/ml, *p* < 0.05).

Significant differences among treatment groups were only observed in IL-6 and TNF-α expression. Concentrations of IFN-γ, IL-10, IL-17A, IL-2 and IL-4 were all at or below the theoretical limit of detection of the BD array in all groups (data not shown).

### FITC-conjugated S. aureus are localised in the olfactory mucosa and periphery of the olfactory bulb

**Figure 3:**
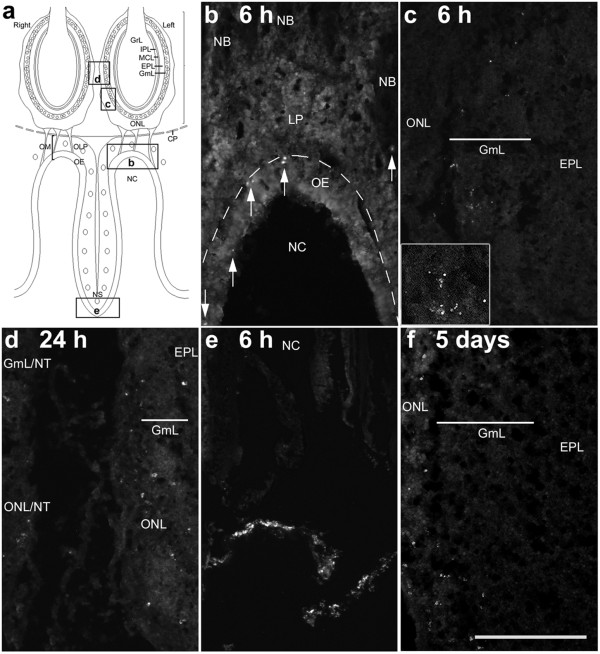
**(A) Line drawing of transverse section of olfactory bulbs and mucosa shows the locations from which micrographs (B-E) were obtained.** At 6 h post-inoculation, FITC-conjugated bacteria (*arrows*) are present in the olfactory epithelium (*OE*) and olfactory nerve bundles (*NB*) in the lamina propria (*LP*; **B**) and in the olfactory nerve layer (*ONL*) and glomerular layer (*GmL*) of the olfactory bulb (**C**). *Inset* in **c** shows aggregates of bacteria. At 24 h post-inoculation, there appear to be more FITC-conjugated bacteria in the ONL and GmL of the treated side with the corresponding layers of the untreated side (*ONL/NT*, *GmL/NT*) showing the presence of a small number of bacteria (**D**). Numerous FITC-conjugated *S. aureus* were located near the nares (**E**). At 5 days post-inoculation, FITC-conjugated bacteria are restricted largely to the ONL (**F**). *NC* = nasal cavity; *EPL* = external plexiform layer. *Scale bar* = 200 μm (**B,C,D,F**), 500 μm (**E**), 100 μm (*inset* in **C**).

At 6 and 24 h after bacterial inoculation into the Triton-X 100 washed nasal cavity, FITC-labelled *S. aureus* could be localised to both the olfactory mucosa and bulb (Figure 3A-C). In the olfactory mucosa, FITC labelling was observed mainly in the olfactory epithelium and in rare instances in nerve bundles within the lamina propria. In the olfactory bulb, fluorescence was observed in the glomerular and olfactory nerve layers. The aggregates of punctate fluorescent label observed in the olfactory bulb (inset in Figure 3C) suggested that the *S. aureus* was most likely to be intracellular. The majority of fluorescent labels were found on the ipsilateral side of bacterial inoculation, although some could be found in the glomerular and olfactory nerve layer of the contralateral bulb (Figure 3D). On the whole, the number of FITC-labelled *S. aureus* localised to olfactory tissues was relatively low. Numerous FITC-labelled *S. aureus* were located near the nares at 6 h (Figure 3E), suggesting that a large number of bacteria were being expelled. Five days after bacterial inoculation, some FITC- labelling remained in the olfactory mucosa, the olfactory nerve and glomerular layers of the ipsilateral olfactory bulb (Figure 3F). At no time was FITC labelling observed in the deeper layers of the olfactory bulb.

### Transmission electron microscopy of S. aureus in olfactory mucosa

**Figure 4:**
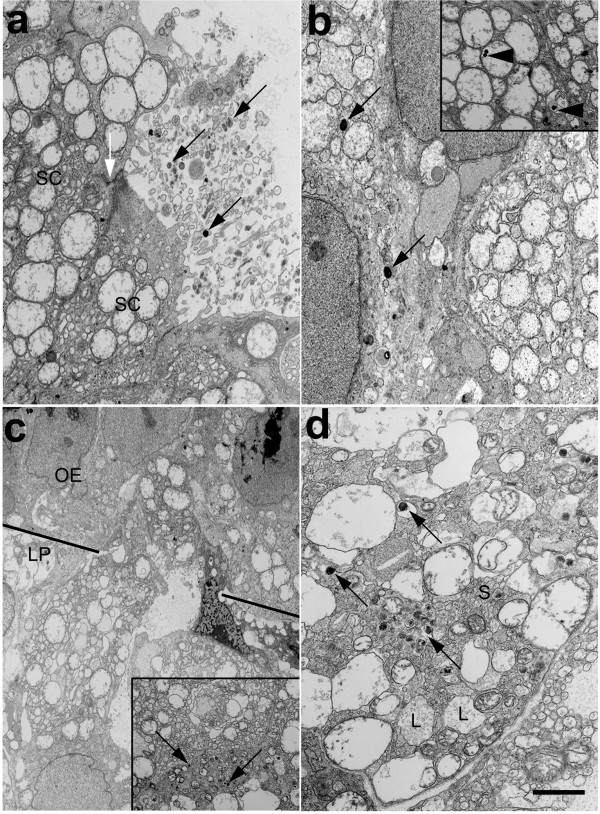
**Ultrastructural distribution of*S. aureus*6 h following nasal inoculation.** Bacteria (*black arrows*) are present on the surface of the epithelium. Junctional complex (*white arrow*) between supporting cells (*SC*) remain intact (**A**). Bacteria may be located in the cytosol (*arrows*; **B**) or within vacuoles of supporting cells (*arrowheads*; *inset* in **B**). Highly vesiculated cells resembling macrophages are observed straddling the olfactory epithelium (*OE*) and lamina propria (*LP*; **C**). Bacteria are present in the vesicles of some macrophages in the lamina propria (*arrows* in *inset* of **C**) or more rarely in olfactory nerve bundles (*arrows*) that are comprised of large (*L*) and small (*S*) unmyelinated axons enveloped by cytoplasmic processes of olfactory ensheathing cells (**D**). *Scale bar* = 2 μm (**A,B,C,D**); 6 μm (*inset* in **C**).

Ultrastructural observations at the 6- and 24-h time points yielded similar results. Up to 24 h after inoculation of *S. aureus* into the nasal cavity previously washed with Triton-X 100, some bacteria remained on the surface of the epithelium (Figure 4A). The junctional complex between supporting cells remained intact and no bacteria were observed extracellularly in the olfactory epithelium. This suggested that *S. aureus* was unlikely to have accessed the lamina propria *via* spaces between epithelial cells. Instead, endocytosed *S. aureus* was localized either in vesicles or in the cytosol of supporting cells (Figure 4B). Highly vesiculated cells resembling macrophages were observed straddling the olfactory epithelium and lamina propria (Figure 4C), some of which in the lamina propria were shown to have endocytosed bacteria (inset in Figure 4C). *S. aureus* was present in a small number of axon bundles (Figure 4D), most likely olfactory rather than trigeminal nerves given that the axons were all unmyelinated.

### Number of iNOS-positive cells in the olfactory mucosa and bulb is increased significantly following exposure to S. aureus

**Figure 5:**
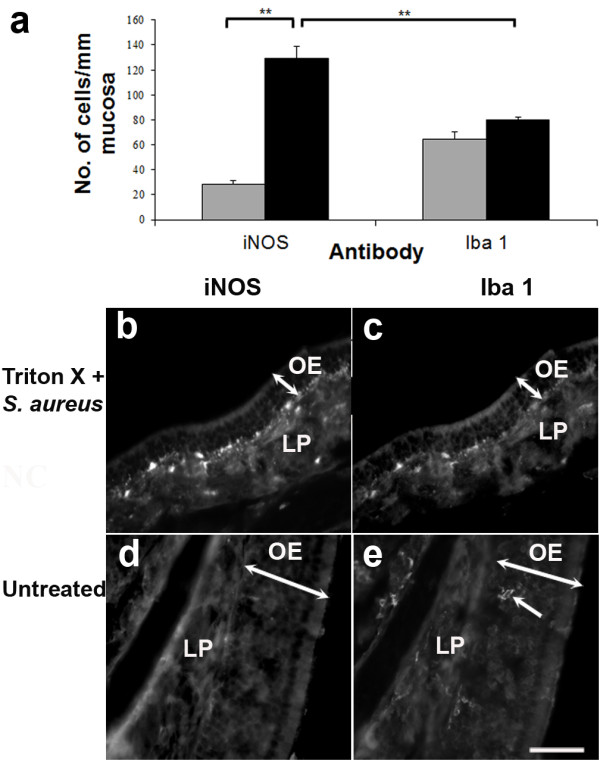
**Density of iNOS- and Iba1-positive cells in the normal (*grey bars*) and bacteria-challenged (*black bars*) olfactory mucosa (A).** The density of iNOS-positive cells following bacterial exposure is significantly more than that in the unperturbed olfactory mucosa, increasing from 28 ± 2 to 129 ± 10 cells/mm mucosa. This resulting high density of iNOS-positive cells is greater than the density of Iba1-positive cells, indicating that a proportion of the iNOS-positive cells are not macrophages. *Error bars* represent SEM. **p < 0.01. The iNOS- and Iba1-positive cells are predominantly located in the lamina propria (*LP*), close to the olfactory epithelium (*OE*; **B**, **C**). In the normal, intact olfactory mucosa (**D,E**), Iba1-positive cells can occasionally be observed in the olfactory epithelium (*arrow* in **E**). *Scale bar* = 100 μm.

**Figure 6:**
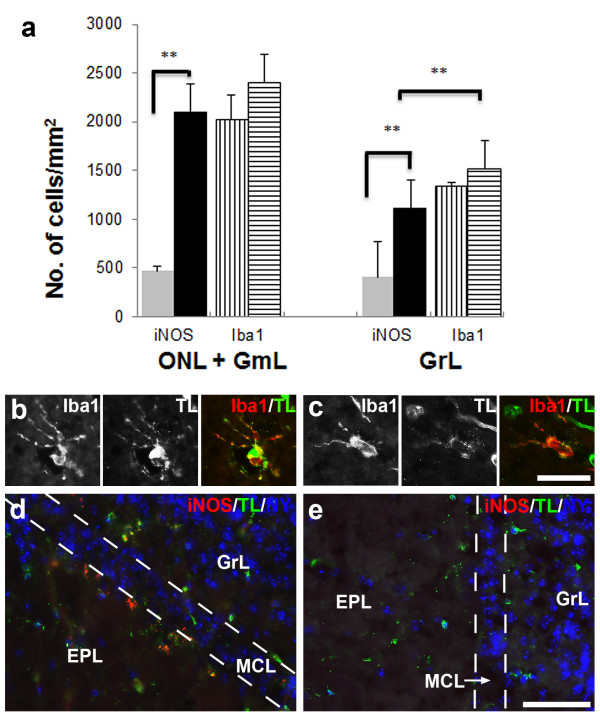
**Density of iNOS- and Iba1-positive cells in the olfactory nerve (*ONL*), glomerular (*GmL*) and granule layers (*GrL*) in the unperturbed and bacterial challenged olfactory bulbs (A).** These layers show a significant increase in the number of iNOS-positive cells in response to bacterial challenge (*black bars*) compared to those in the untreated control olfactory bulb (*grey bars*), 4.5 fold increase in the ONL + GmL and 2.8 fold increase in the GrL. However there is no significant increase in the number of Iba1-positive cells between the untreated olfactory bulb (*bars* with *vertical lines*) and *S. aureus*-exposed olfactory bulb (*bars* with *horizontal lines*). *Error bars* represent SEM. **p < 0.01. Fluorescence microscopy shows activated microglia with hypertrophied cell bodies and ramifying processes, and demonstrating co-localisation of Iba1 and tomato lectin (*TL*) in *S.aureus*-exposed olfactory bulb (**B**, **C**). Microglia (*green*: TL) expressing iNOS (*red*) in the vicinity of the mitral cell layer (*MCL*) in *S.aureus*-exposed olfactory bulb (**D**). No apparent staining for iNOS is observed in a similar area in this saline-treated olfactory bulb (**E**). *NY* = Nuclear yellow; *EPL* = external plexiform layer. *Scale bar* = 25 μm (**B,C**), 50 μm (**D,E**).

Given that FITC-conjugated *S. aureus* waspresent in the glomerular layer of the olfactory bulb 24 h after bacterial exposure, quantitative analysis was conducted to establish whether this process induced nitric oxide production in the related regions. Although a previous study from our laboratory showed that some OECs in the lamina propria were induced to express iNOS in response to bacterial exposure, we did not quantify the relative increase of iNOS-expressing cells or the contribution of macrophages to the iNOS-expressing population [6]. Here we show that the number of iNOS-positive cells in the olfactory mucosa and bulb was significantly increased following bacterial exposure (*p* = 0.01; Figure 5A and 6A). The density of iNOS-positive cells increased 4.4 fold (from 28 ± 2 to 129 ± 10 cells/mm mucosa) in the olfactory mucosa, 4.5 fold (from 468 ± 46 to 2,102 ± 196 cells/mm^2^) in the olfactory nerve and glomerular layers, and 2.8 fold (from 403 ± 21 to 1,113 ± 181 cells/mm^2^) in the granule cell layer. In the damaged olfactory mucosa exposed to *S. aureus*, iNOS-positive cells were present predominantly in the lamina propria close to the olfactory epithelium (Figure 5B). About 62% of these cells were positively immunolabeled with Iba1 (Figure 5A5C), indicating that they were macrophages present either in the lamina propria or olfactory epithelium (Figure 5), while the rest were likely to be OECs, as demonstrated in our previous study [6]. Although the density of activated cells, as represented by iNOS-immunoreactivity, was significantly increased in the *S. aureus*-treated tissue, the density of macrophages was not significantly increased compared to untreated tissue (Figure 5A).

In the olfactory bulb, there was also a general increase in the density of iNOS-expressing cells in the *S. aureus*-treated mice, as shown by the quantitative analysis done on the olfactory nerve, glomerular and granule cell layers (Figure 6A). Although no significant increase in Iba1-positive cells was observed in the olfactory bulb, they had an activated morphology, as evidenced by the hypertrophied cell bodies and ramified processes. Further confirmation that the Iba1-positive cells were microglia was provided by their positive binding to tomato lectin with variable intensities (Figure 6B, C). In the *S. aureus*-exposed olfactory bulbs, iNOS-expressing microglia were present not just in the peripheral layers, but also in the vicinity of the mitral cell layer (Figure 6D). In comparison, saline-treated olfactory bulbs generally showed sparse or no immunoreactivity for iNOS (Figure 6E), similar to untreated olfactory bulbs (data not shown).

## Discussion

This study demonstrates that damage to the olfactory epithelium compromises the protective barrier and allows bacteria to gain access to the outer layers of the olfactory bulb as rapidly as 6 h following exposure. Infiltration of *S. aureus* into the nasal tissues is accompanied by upregulation of IL-6, TNFα and iNOS expression. In the olfactory mucosa the cellular immune defence is provided by macrophages and olfactory ensheathing cells, while microglia constitute the major defensive cell in the olfactory bulb. *S. aureus* is restricted to the olfactory nerve and glomerular layers of the olfactory bulb up to 5 days after bacterial exposure.

### Biological significance of cytokines in the immune response of olfactory tissue

Cytokines are humoral components in innate immunity that are vital in the initiation and regulation of an appropriate immune response [19]. In this study expression of IL-6 and TNF-α was found to significantly increase at both 6 and 24 h after bacterial inoculation in mice whose olfactory epithelium had been previously damaged. The results are consistent with an earlier study in which elevated IL-6 and TNF-α mRNA levels in the murine olfactory mucosa and bulb were demonstrated at 24 h following intranasal inoculation with Satratoxin G, a mycotoxin that damages the olfactory epithelium [20]. Signalling *via* cytokines such as IL-6 and TNF-α, produced by macrophages as a result of bacterial detection, has been shown to be vital in the initiation of immune responses to directly remove the infecting agent [21,22].

TNF-α, being a pro-inflammatory cytokine, induces a number of different responses that can potentially exacerbate tissue damage. However, without it, brain abscesses induced by *S. aureus* persist, as evidenced by increased bacterial burdens [23]. Therefore, tight regulation of TNF-α expression needs to occur to maintain a balance between destruction of normal tissue and removal of potentially devastating bacteria. Pituitary adenylate cyclase-activating peptide (PACAP) is a protein that is produced by OECs and supporting cells of the olfactory epithelium [24] that has been shown to protect against TNF-α induced cell death in olfactory neurons [25]. It is possible that PACAP is produced locally by olfactory ensheathing cells and sustentacular cells in order to mitigate the damaging effects of TNF-α in the process of bacterial elimination. Furthermore the IL-6 receptor is known to be expressed transiently by OECs following neuronal cell death in response to bulbectomy [26], suggesting that it may be a primary target of IL-6, including that produced by OECs themselves. IL-6/IL-6R binding in OECs may be protective by inducing nuclear translocation of STAT transcription pathways, thereby activating anti-apoptotic pathways [27]. The survival of OECs would have particular importance in compromised olfactory tissue as these cells have a vital contribution to olfactory neuronal regeneration and innate immunity [28,29].

Studies on macrophages and OECs have shown that NFκB nuclear translocation occurs in response to bacteria and pathogen-associated molecular patterns, resulting in iNOS transcription and nitric oxide production [6,30]. Though this may be a direct effect of bacteria on innate immune cells, nitric oxide may also be induced by TNF-α binding to mTNFR1, which then activates a number of cellular pathways, including the activation and nuclear translocation of NFκB [31,32]. NFκB activation also induces IL-6 transcription [33,34]. In our study, a significant upregulation of TFN-α in olfactory tissues at the 6-h time point preceded a significant upregulation of IL-6 at the 24-h time point. A possible explanation is that while TNF-α is produced to recruit factors that remove *S. aureus*, it also induces the production of IL-6. In this study, at 24 h after bacterial infection, IL-6 immunoreactivity was present in supporting cells, olfactory ensheathing cells, fibroblasts, endothelial cells and innate immune cells. IL-6 has been previously shown to be expressed by macrophages and olfactory ensheathing cells [26,35] as well as endothelial cells and fibroblasts in inflammatory conditions [36,37]. IL-6 mediates neutrophil responses (Dalrymple *et al.*, 1995; Dalrymple *et al.*, 1996) and is also known to enhance neuron survival (Toulmond *et al.*, 1992; Loddick *et al.*, 1998), and therefore may be neuroprotective with regard to olfactory neuronal survival. Therefore, cytokines such as TNF-α and IL-6 could work in synergy to maintain olfactory tissue homeostasis while removing the bacterial infection.

### Infiltration of bacteria in the primary olfactory pathway

This is the first study to show that bacteria in the nasal cavity were able to reach the olfactory bulbs in as little as 6 h. Previous studies had used longer time points to assess bacterial invasion in the nasal connective tissue and olfactory bulb [5,6]. In our study, *S. aureus* was localised ultrastructurally to supporting cells of the olfactory epithelium, macrophages and a small number of olfactory nerve bundles. Although previous studies have indicated that olfactory ensheathing cells have anti-bacterial properties by their expression of Toll-like receptors (TLRs), nuclear translocation of nuclear factor kappa B and production of nitric oxide in the presence of bacteria [6,16,29], these mechanisms in combination with macrophages of the lamina propria may not be entirely effective in preventing infiltrating bacteria from accessing the olfactory bulb. Therefore, under such circumstances, olfactory ensheathing cells could act as a host for invading bacteria [38].

Mitral cells were the major population of cells in the olfactory bulb showing immunoreactivity for IL-6, with more diffuse staining in the external plexiform layer. The immunostaining pattern suggests that the mitral cell dendrites extending through the external plexiform layer and terminating in the glomerular layer were stimulated by *S. aureus*, which then induced upregulation of IL-6 expression. Previous studies involving intranasal inoculation of mouse hepatitis virus revealed that viral RNA could be detected in mitral cells 5 days after, eventually resulting in a significant decrease in the mitral cell population [39]. This suggested that mitral cells are vulnerable to infection from peripheral sources. Therefore, expression of IL-6 may be upregulated in response to bacteria as a protective mechanism to inhibit infection of the cells.

### Activation of microglia in the olfactory bulb

In this study the presence of *S. aureus* in the olfactory nerve and glomerular layers was accompanied by the appearance of activated microglia throughout the olfactory bulb, suggesting that although not in direct contact with the pathogens, an intercellular sensing mechanism induces rapid activation. A recent study has suggested that olfactory bulb microglia may be distinct from microglia from other parts of the CNS by having a lower activation threshold [40]. Lalancette-Hébert and co-workers showed that following induction of focal cerebral ischemia by occlusion of the left middle cerebral artery, a significant induction of TLR2 signals in microglia of the olfactory bulbs was detected 6 h later, which preceded any increase in TLR2 signals in and around the ischaemic site. Furthermore, when lipopolysaccharide, a component of gram-negative bacterial cell wall, was injected into the nasal cavity, upregulation of TLR2 signals in the olfactory bulb, particularly in the glomerular layer, was similarly detected at 6 h, and this spread to other parts of the brain by 24 h [40]. These observations suggest that microglia of the olfactory bulbs have a more alert, activated phenotype than microglia in the rest of the CNS. Thus, they play a key role in the regulation of inflammatory processes in the brain. In this regard, the finding in our study that *S. aureus* was restricted to the outer nerve and glomerular layers at 5 days after inoculation is further support that the activated microglia of the olfactory bulb have a role in neutralising bacterial pathogens and are likely to be the key player that prevents pathogens in the periphery of the olfactory bulb from further migration into the CNS. In this regard, olfactory microglia clearly have a beneficial effect in safeguarding the CNS from harmful pathogens.

However, the immunomodulating property of olfactory bulb microglia could act as a modifier of therapeutic agents administered intranasally. Several studies have shown the efficacy of the primary olfactory pathway as a route of entry for growth factors and hormones into the CNS [41-43]. Given their higher basal activity, it is possible that intranasally administered compounds, both natural and synthetic, could trigger activation of olfactory bulb microglia, thus releasing a host of molecules including pro-inflammatory cytokines that have wide-ranging effects not only on surrounding cells, but also on the administered compound [44,45]. For example, elevated secretory response from the microglia could change the internalisation and transport, biodistribution and perhaps ultimately the therapeutic potential of the administered compound. Thus, the activity of olfactory bulb microglia warrants further investigation, in terms of their physiological role in protecting the brain from pathogenic insults as well as their interaction with nasally administered exogenous compounds.

## Conclusions

Bacteria are able to penetrate the immunological defence of the compromised olfactory mucosa and infiltrate the olfactory bulb within 6 h even though a pro-inflammatory profile is mounted. Activated microglia may have a role in restricting bacteria to the outer layers of the olfactory bulb. These findings have implications with regard to susceptibility to CNS infections in the aftermath of damage to the nasal lining.

## Competing interests

The authors declare that they have no competing interests.

## Authors’ contributions

RH carried out the olfactory epithelial ablation, cytokine array, fluorescence imaging and cell culture, and assisted in the preparation of figures. JH carried out the olfactory epithelial ablation and immunofluorescence, and assisted in the preparation of figures. KPC participated in olfactory epithelial ablation and transmission electron microscopy. JC participated in transmission electron microscopy. AW participated in the design and data analysis of the study. MIC conceived of the study, participated in the design, assisted in some experiments and drafted the manuscript. All authors read and approved the final manuscript.
